# In-person and online sensory wellbeing workshop for eating disorders: updated case series

**DOI:** 10.1186/s40337-023-00834-8

**Published:** 2023-07-13

**Authors:** Zhuo Li, Victoria Holetic, Jessica Webb, Dimitri Chubinidze, Sarah Byford, Kate Tchanturia

**Affiliations:** 1 Department of Psychological Medicine, Institute of Psychiatry, Psychology, and Neuroscience, King’s College London, London, UK; 2 National Eating Disorders Service, South London and Maudsley NHS Foundation Trust, London, UK; 3 Tbilisi Ilia State University, Tbilisi, Georgia; 4 King’s Health Economics, Health Service and Population Research Department, Institute of Psychiatry, Psychology and Neuroscience, King’s College London, London, UK

**Keywords:** Eating disorder, Sensory system, Sensory wellbeing, Group, Workshop

## Abstract

**Background:**

A one-off sensory wellbeing workshop has been developed to help patients with eating disorders (ED) manage sensory sensitivities. The aim of this study was to evaluate and compare the outcomes of the workshop in online versus face-to-face (F2F) formats among a sample of patients with ED.

**Methods:**

Cumulative link models were applied to the outcome measures (awareness of sensory wellbeing, awareness of strategies to enhance sensory wellbeing, and confidence in managing sensory wellbeing) to test the differences between online and F2F workshops. Participants’ ratings of usefulness of the workshop were also compared between online and F2F workshops.

**Results:**

A total of 14 workshops (4 online and 10 F2F) were run from 2020 to 2023. All participants reported significant and substantial improvements in all outcome measures. There was no significant difference in outcomes between online and F2F workshops. The majority of patients rated the workshops as useful.

**Conclusions:**

Both online and face-to-face formats of the sensory workshop led to improvement in sensory wellbeing management for patients with ED. Future studies are warranted to test the impact of the workshop on ED treatment outcomes.

**Supplementary Information:**

The online version contains supplementary material available at 10.1186/s40337-023-00834-8.

## Abstract (plain language summary)

People with eating disorders often have sensory issues, which can include being too sensitive to some senses (hearing, smell or taste, for example) or not sensitive enough. Explaining how the sensory system works and developing helpful strategies to manage sensory difficulties could be beneficial in the process of therapy. To try and support this, clinicians and researchers designed a sensory wellbeing workshop to help people become more aware of their sensory wellbeing and teach them strategies to manage their sensory wellbeing. The workshop can be delivered online or face-to-face. This study examined the feedback for online and face-to-face workshops. We found that both formats were helpful for people with eating disorders. We also discuss possible ways to develop and test the workshop further in order to better support patients with sensory difficulties.

**Supplementary Information:**

The online version contains supplementary material available at 10.1186/s40337-023-00834-8.

## Introduction

Sensory disturbances in patients with eating disorders (ED), particularly anorexia nervosa (AN), have been widely studied in recent years [9, 12]. Some argue that sensations are commonly muted in individuals with AN, leading to increased reliance on other external cues and rules to regulate eating behaviour [19], whereas others have identified sensory hypersensitivities in AN [30] which can lead to sensory avoidance. For example, studies have found that patients with AN had lower olfactory threshold [25] and increased smell capacity [7, 20] than controls, which could make certain sensations (e.g. strong smell of food) exceedingly intolerable. Moreover, difficulties in interpreting and tolerating these sensations can affect emotional regulation, as individuals may not be able to appropriately guide emotional reactions using body signals [16]. These individuals may then use the ED as a maladaptive coping strategy for negative emotions.

Among individuals with ED, research has also identified a subgroup with a comorbidity of autism that have a more complex presentation [3, 13]. There have been consistent findings of a relationship between sensory processing and eating behaviours in autistic individuals [17], as well as association between autism, sensory processing, and illness severity in individuals with ED [22]. Sensory difficulties are present in 90% of children and adults with autism [14], which could exacerbate sensory issues when comorbid with ED. Indeed, patients with both conditions exhibit heightened sensory sensitivities in areas of smell, taste, vision, and texture [10, 17], leading to maladjustment to standard treatment settings and active avoidance of certain foods [15].

To support patients with hyper- or hypo-sensitivities, it is important to provide a space, psychoeducation and materials to explore their sensory needs. Therefore, a one-off sensory wellbeing workshop was developed by the PEACE (Pathway for Eating disorders and Autism developed from Clinical Experience) pathway [27], (for details of the pathway: www.peacepathway.org) based on previous research as well as perspectives of people with lived experience of sensory sensitivities [10, 11]). This workshop combines psychoeducational materials and practical activities, with the aim to improve sensory awareness and provide sensory management strategies to support sensory wellbeing. We previously conducted a pilot evaluation of the sensory workshop [28] to examine its feasibility and discuss possible areas for development of the workshop. Significant improvement was found in all post-workshop measures with large effect sizes, indicating possibility for the workshop to be delivered as part of ED treatment. Areas of improvement were also identified, including the need for longer workshop duration, more activities, collaboration across clinical services, and possibly introducing a follow up session. Given the limitations of sample size in the pilot study, we have since organised more workshops that are longer in duration, delivered online and in person across clinical services, offering enriched psychoeducational content and activities based on the feedback we received from pilot workshops.

Psychological work should be based in evidence to ensure they are of significant clinical benefit for patients [24]. Therefore, this follow-up study aims to: 1) generate more practice-based experience for the sensory workshop by conducting a case series with an increased sample size; and 2) further investigate the impact of workshop format by comparing the outcomes of face-to-face and online workshops.

## Methods

### Participants

All participants of the study were adult patients with an established DMS-5 [1] diagnosis of ED, admitted to the South London and Maudsley NHS Foundation Trust (SLaM) National Eating Disorder Service and South West London and St George’s Mental Health NHS Trust Specialist Eating Disorder Service. Participants who did not complete the pre-workshop or post-workshop measures were excluded from analysis.

### Measures

All participants were given a pre-workshop questionnaire to complete at the start (T1) of the workshop, and a post-workshop questionnaire at the end (T2). Full questionnaires can be found in the Additional file 1: Appendix. The pre- and post-workshop questionnaires consisted three Likert scale items asking participants to rate their awareness of their own sensory wellbeing (“How aware are you of your sensory wellbeing?”), awareness of strategies to enhance sensory wellbeing (“How aware are you of the strategies to enhance your sensory wellbeing?”), and their confidence in managing their own sensory wellbeing (“How confident do you feel to manage your sensory wellbeing?”). The post-workshop questionnaire contained an additional question asking participants to rate the usefulness of the workshop (“How useful was this sensory workshop?”). All questions used a 5-point Likert scale ranging from 1 (“Not aware/confident/useful at all”) to 5 (“Really aware/confident/useful”). By comparing participant responses before and after the workshop, we aimed to evaluate the change in participants’ self-awareness and abilities to manage their sensory wellbeing. Furthermore, by including a question on usefulness in the post-workshop questionnaire, we can gauge participant satisfaction which is valuable for the workshop’s future refinement.

### Procedure

Detailed procedure and protocol of the sensory workshop can be found in the pilot evaluation by Tchanturia et al. [28]. In brief, the workshop was advertised to all patients in the services through poster and community meetings. Attendance was voluntary. The in-person workshop begins with psychoeducation of the different senses and discussion of sensory experiences, followed by two exercises: an exploration of different materials to identify one’s own sensory preferences, and a do-it-yourself (DIY) activity of creating a sensory item of choice, for example a glitter jar, a scented hand cream or choose materials which have soothing effect when touched (fluffy, firm, soft textiles). Take home materials such as further psychoeducational worksheet and tools to communicate sensory preferences were also provided. At the start and end of the workshop, participants were asked to complete the pre (T1) and post (T2) workshop questionnaires. The in-person workshops lasted for a duration of two hours and were facilitated by two to three members of clinical staff.

During the COVID-19 pandemic, the sensory workshop was adapted for online delivery via Microsoft Teams and run from December 2020 to April 2022. The psychoeducational content was adapted to a PowerPoint presentation, and discussions were facilitated online. An interactive presentation software, named Mentimeter, was used to facilitate discussions. Following psychoeducation, the Mentimeter tool was utilised to prompt participants to write and post answers freely to two questions: ‘what senses are comforting to me?’ and ‘what senses bother me?’, and the answers were discussed as a group. For the DIY element, participants were encouraged to identify and prepare their own sensory items for the exercises. Participants who did not have items at hand would discuss and describe the sensory items they found helpful. Electronic versions of the pre- and post-workshop feedback questionnaires were distributed, and the take home materials were circulated after the workshop via e-mail. The online workshop ran for one and a half hours, shorter than the in-person workshop as material preparation time was deducted, and was facilitated by two to three members of the clinical team.

Overall, the two workshop formats differ most significantly in the provision of materials for the practical element. The in-person workshop includes a hands-on activity of making a sensory item using materials provided by facilitators, whereas in the online format participants were required to bring or discuss their favorite sensory items. To ensure participant engagement, break out rooms of smaller groups were used in online workshops, with one facilitator in each break out room leading the discussion.

### Analysis

Within-group analysis was conducted using Wilcoxon signed ranks tests to examine improvement on each measure in in-person and online workshops individually. Furthermore, between-group analysis was conducted to investigate the effect of workshop format for each outcome measure (awareness of sensory wellbeing, awareness of strategies to enhance sensory wellbeing, and confidence in managing sensory wellbeing) using cumulative link mixed models fitted with the Laplace approximation, the most popular class of ordinal regression models, due to its suitability for repeated measures ordinal data analysis [5]. Group (online vs face-to-face) and time (T1 and T2) and the interaction between them were included as explanatory variables and individual identity as random variable. In addition, for the ‘usefulness’ measure which is only answered once at post-workshop, a Mann-Whitney U test is used to compare between online and face-to-face workshops. Data were analysed using IBM SPSS software (Version 28) and the clmm function in the ordinal package for R [21].

## Results

In total, 14 workshops (4 online and 10 face-to-face) including 86 participants (26 online and 60 face-to-face) were run from February 2020 to May 2023. The number of participants for each workshop ranged from 2 to 10. Eighty-one patients (23 online and 58 face-to-face) submitted anonymous feedback at T1 and/or T2. Among them, feedback was partly missing (in either pre- or post-workshop measure) for 10 (43.5%) online participants and 5 (8.6%) face-to-face participants. These participants were excluded by case from analysis. As a result, a total of 66 valid responses (13 online and 53 face-to-face) were included in the analysis. Their baseline characteristics are summarised in Table 1. There was no significant difference between online and face-to-face participants in their baseline characteristics.

**Table 1:** Baseline characteristics of participants in online and face-to-face (F2F) workshops

	Online (N = 13)	F2F (N = 53)
Age (years), mean (SD)	23.2 (4.1)	25.8 (7.9)
Missing	2 (15.4%)	7 (13.2%)
Diagnosis, n (%)		
AN restrictive	9 (69.2%)	32 (60.4%)
AN binge-purge	2 (15.4%)	9 (17%)
AN atypical	0	2 (3.7%)
Bulimia nervosa	0	1 (1.9%)
Binge eating disorder	0	1 (1.9%)
Other specified feeding and eating disorder (OSFED)	1 (7.7%)	1 (1.9%)
Missing	1 (7.7%)	7 (13.2%)
Gender, n(%)		
Female	13 (100%)	45 (84.9%)
Male	0	0
Other	0	1 (1.9%)
Missing	0	7 (13.2%)
BMI on admission, mean (SD)	16.35 (2.45)	15.35 (4.63)
Missing, n(%)	2 (15.4%)	7 (13.2%)
Ethnicity		
White British	12 (92.3%)	36 (67.9%)
White Irish	0	1 (1.9%)
White other	0	3 (5.7%)
Black Afro-Caribbean	0	2 (3.8%)
Black British	0	1 (1.9%)
Asian (Indian)	1 (7.7%)	0
Mixed	0	3 (5.7%)
Missing	0	7 (13.2%)

**Fig. 1:**
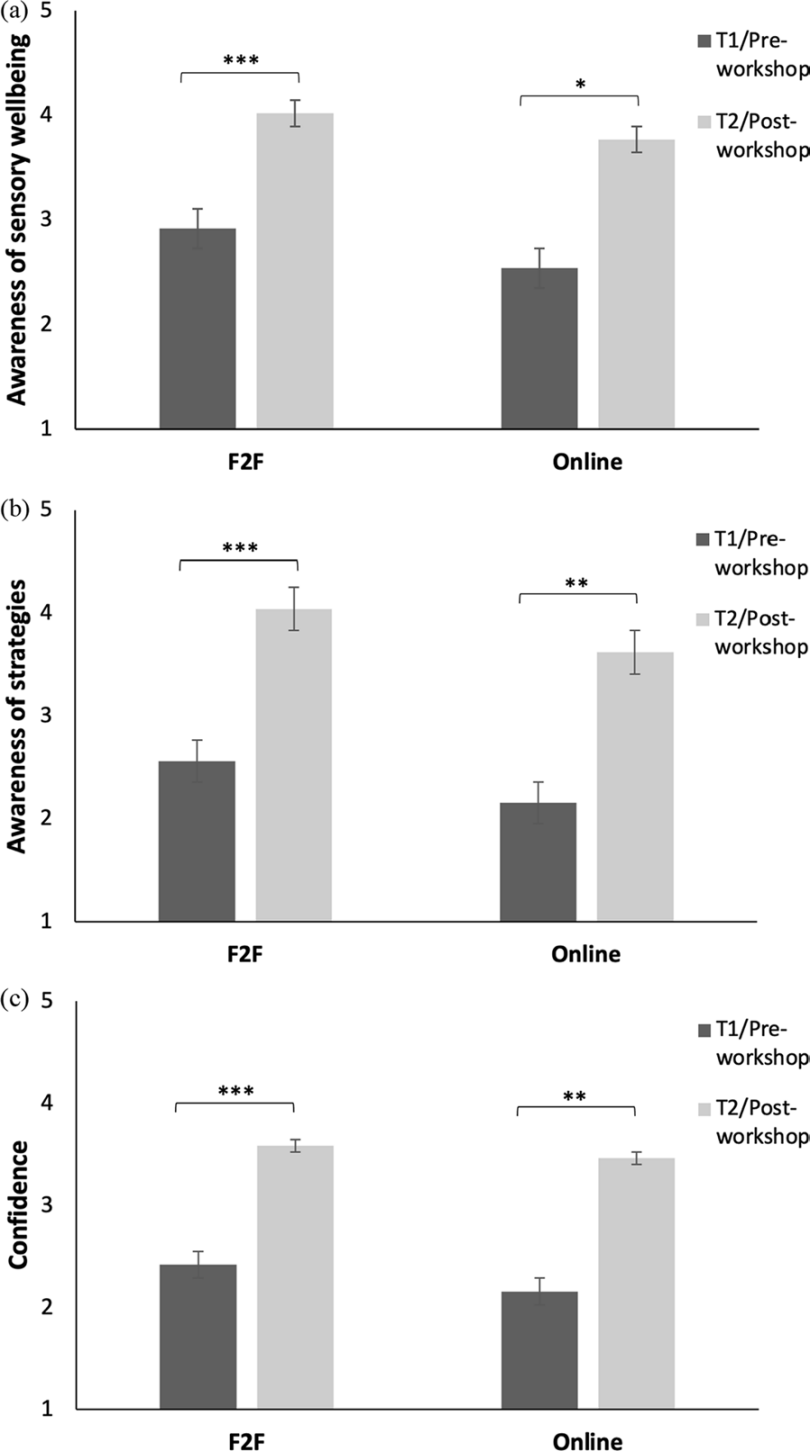
Comparison of face-to-face (F2F) and online workshop scores for **a** Awareness of sensory wellbeing, **b** Awareness of strategies to enhance sensory wellbeing and **c** Confidence in managing sensory wellbeing. *Note* *p < 0.05, **p < 0.01, ***p < 0.001

Outcomes are summarised in Table 2 and visualised in Fig. 1a–c. Both face-to-face and online workshops saw statistically significant improvement in all measures with large effect sizes.

**Table 2:** Summary of pre-workshop (T1) and post-workshop (T2) participant feedback

Measure	Workshop format	T1		T2		Difference		
		M	SD	M	SD	*Z*	*p*	Cohen’s *d*
Awareness of sensory wellbeing	F2F	2.92	1.03	4.00	0.76	− 5.21	< .001	1.08
	Online	2.54	1.13	3.77	0.73	− 2.55	.011	0.95
Awareness of strategies	F2F	2.49	1.12	4.02	0.69	− 5.65	< .001	1.25
	Online	2.15	0.90	3.62	0.77	− 3.13	.002	1.88
Confidence	F2F	2.38	0.88	3.51	0.80	− 5.56	< .001	1.15
	Online	2.15	1.14	3.46	0.97	− 2.85	.004	1.27

Table 3 shows the results of cumulative link models for all measures. Time had a significant effect on awareness of sensory wellbeing (*p* = 0.019), awareness of strategies to manage sensory wellbeing (*p* < 0.001), and confidence in managing sensory wellbeing (*p* = 0.038), suggesting that participants improved significantly on all measures. Neither workshop format nor the interaction between time and workshop format had a significant impact on the outcomes, suggesting that improvement on the outcomes was not significantly different between in-person and online workshops.

**Table 3:** Summary of cumulative link models for all workshop measures

	Awareness of sensory wellbeing			Awareness of strategies			Confidence		
	Coefficient	SE	*p*	Coefficient	SE	*p*	Coefficient	SE	*p*
Time (T1 vs. T2)	2.66	1.14	.019*	3.87	1.13	< .001***	2.36	1.14	.038*
Format (F2F vs. online)	− 0.85	0.76	.266	− 0.66	0.64	.302	− 0.97	0.79	.224
Time x Format	0.14	0.87	.874	− 0.39	0.81	.629	0.79	0.89	.372

^*^p < 0.05, **p < 0.01, ***p < 0.001

In terms of post-workshop ratings of usefulness, 51 (96.2%) participants of the face-to-face workshop and 12 (92.3%) participants of the online workshop rated it 3 (“Quite useful”) to 5 ("Really useful”). The mean rated usefulness was 4.01 for face-to-face and 3.77 for online workshops. Mann-Whitney test showed that the two workshop formats did not differ significantly in reported usefulness (*U* = 265, *p* = 0.366).

## Discussion

In this paper, we provide updated results for the sensory wellbeing workshop since the publication of its pilot evaluation [28]. Overall, the results are in line with the original paper, with participants reporting significant and substantial improvements in all measures (awareness of sensory wellbeing, awareness of strategies to manage sensory wellbeing, and confidence in managing sensory wellbeing) at post-workshop. Our results contribute to the growing body of literature that attests to the positive patient experiences and outcomes associated with group therapies [18, 23, 26], and continues to demonstrate the feasibility of incorporating group workshops as adjunct elements within ED treatment programs [28].

Furthermore, our results provide support for online provision, with no difference in outcomes between workshops delivered in person and online. This finding highlights the adaptability of the workshops and their potential for broader dissemination. However, it is essential to consider the practical differences between the formats. In-person workshops create a more hands-on and interactive environment, which facilitates better demonstrations of sensory items and encourages social interaction among participants. Conversely, online workshops may face challenges in achieving the same level of engagement and interaction as face-to-face sessions. Future studies comparing between the two workshop formats on the level of participant engagement are warranted. Despite these potential challenges, the online format offers increased accessibility and flexibility, particularly for those who may encounter barriers to attending in-person workshops. Furthermore, the following recommendations may help enhance the delivery of online sensory wellbeing workshops: (1) Streamlined material acquisition: It would be beneficial to offer pre-assembled material packs for participants who may encounter challenges in obtaining the necessary items themselves. This approach ensures that all attendees have the requisite resources for the online workshop. (2) Workshop automation: to aid item demonstrations in the online workshops, we suggest incorporating pre-recorded content, interactive tools, or self-paced activities in the workshop. This approach will foster a more streamlined and efficient experience while preserving engagement and interactivity. (3) Introducing breaks: As the focus on psychoeducation in online workshops can be mentally taxing for participants, regular short breaks could be introduced into the workshop, for example in between the psychoeducation and discussion sessions.

Research is sparse when investigating sensory processing within ED behaviours. However, previous studies have demonstrated that individuals with ED have more sensory disturbances than healthy controls [30]. For example, Gaudio et al. [8] found that individuals with AN may have multisensory impairments regarding their body perception, including both tactile and proprioceptive sensory components. Other studies have demonstrated that individuals with ED may have higher sensory sensitivities or even avoid sensory experiences and appear less able to appropriately identify satiety sensations [6, 19] or recognise internal signals relating to stress such as increased heart rate [29]. A more recent study showed that those with AN had significantly lower sensory registration and seeking behaviour, along with increased sensitivity and sensory avoidance compared to healthy controls [22]. It is worth noting that most of the work have a focus on participants with AN. We have included patients with all EDs in the current study but the majority of patients had AN, which reflects the patient demographics at the ED service. Future studies should consider including different patient groups to investigate the impact of addressing sensory difficulties in patients with bulimia nervosa or binge eating disorder.

Furthermore, understanding subjective body experience and its linkage with emotional awareness and regulation is crucial when challenging ED symptomology and cognitive distortion in patients with EDs. Previous work has linked sensory processing impairment with self-disgust in AN as well as BN [2]. It is important to note that as well as the relentless pursuit for the ‘perfect’ body, patients may also be motivated to maintain disordered eating to alter their body experiences [30]. There is also evidence that individuals with AN have deficits in integrating visual and proprioceptive information, which may contribute to the distorted body image in AN [4]. Therefore, sensory processing difficulties could be a crucial target when addressing the maintenance factors of the illness. Following this early stage evaluation of the sensory workshop, further research is needed, perhaps on a more longitudinal scale, to measure the impact of addressing sensory experience on ED treatment outcomes.

The present study is limited by the sample size for online workshops as well as missing data. Furthermore, feedback was partly incomplete for 43.5% of online participants and 5.9% of face-to-face participants, suggesting that participants of the face-to-face workshops were more likely to fill in the outcome measures than those of the online workshops. Methods for online feedback collection may need to be improved, and findings comparing the two workshop formats therefore need to be interpreted with caution. More rigorous trials of the workshop need to be conducted in the future, incorporating a wider range of outcome measures (including ED symptom measures) as well as a control group to quantify outcomes.

## Conclusion

Both online and face-to-face formats of the sensory workshop led to improvement in awareness of sensory wellbeing and confidence in managing sensory wellbeing for patients with ED. Future studies are warranted to investigate the impact of the workshop on ED treatment outcomes.

## Supplementary Information

**Additional file 1:** Sensory wellbeing workshop feedback survey.

## Data Availability

All data generated or analyzed during this study are available upon request.

## Abbreviations

ED: Eating disorder
AN: Anorexia nervosa
F2F: Face to face
ARFID: Avoidant/restrictive food intake disorder
