# Supplementary material for: In-person and online sensory wellbeing workshop for eating disorders: updated case series
Source: J Eat Disord. 2023 Jul 13;11:117. doi: 10.1186/s40337-023-00834-8 (PMC10347786; doi:10.1186/s40337-023-00834-8)
Supplement: Supplementary file 1 — Additional file 1: Sensory wellbeing workshop feedback survey. [file 40337_2023_834_MOESM1_ESM.docx]

**Appendix: Sensory wellbeing workshop feedback survey**

**Sensory Workshop**

Date:………………… Initials:…………………

**Please complete this section at the end of the sensory workshop:**

(Please indicate the extent to which you agree with each statement by circling the number from 1 to 5)

1. **How aware are you of your sensory sensitives at the end of this workshop?**

| Not aware at all |  | Quite aware |  | Really aware |
| --- | --- | --- | --- | --- |
|  |  |  |  |  |
| 1 | 2 | 3 | 4 | 5 |

1. **How aware are you of the strategies to manage your sensory sensitives as a result of the workshop?**

| Not aware at all |  | Quite aware |  | Really aware |
| --- | --- | --- | --- | --- |
|  |  |  |  |  |
| 1 | 2 | 3 | 4 | 5 |

1. **How confident do you feel to manage your sensory sensitivities following the sensory workshop?**

| Not confident at all |  | Quite confident |  | Really confident |
| --- | --- | --- | --- | --- |
|  |  |  |  |  |
| 1 | 2 | 3 | 4 | 5 |

1. **How useful was this sensory workshop?**

| Not useful at all |  | Quite useful |  | Really useful |
| --- | --- | --- | --- | --- |
|  |  |  |  |  |
| 1 | 2 | 3 | 4 | 5 |

1. **What did you like most about this sensory workshop?**

**……………………………………………………………………………………………………………………………**

**……………………………………………………………………………………………………………………………**

1. **Any other comments?**

**……………………………………………………………………………………………………………………………**

**……………………………………………………………………………………………………………………………**

**Please complete this section at the start of the sensory workshop:**

(Please indicate the extent to which you agree with each statement by circling the number from 1 to 5)

1. **How aware are you of your sensory sensitives?**

| Not aware at all |  | Quite aware |  | Really aware |
| --- | --- | --- | --- | --- |
|  |  |  |  |  |
| 1 | 2 | 3 | 4 | 5 |

1. **How aware are you of the strategies to manage your sensory sensitives?**

| Not aware at all |  | Quite aware |  | Really aware |
| --- | --- | --- | --- | --- |
|  |  |  |  |  |
| 1 | 2 | 3 | 4 | 5 |

1. **How confident do you feel to manage your sensory sensitivities?**

| Not confident at all |  | Quite confident |  | Really confident |
| --- | --- | --- | --- | --- |
|  |  |  |  |  |
| 1 | 2 | 3 | 4 | 5 |
